# Supplementary material for: Registered Report: Neural correlates of thematic role assignment for passives in Standard Indonesian
Source: PLoS One. 2025 May 13;20(5):e0322341. doi: 10.1371/journal.pone.0322341 (PMC12074587; doi:10.1371/journal.pone.0322341)
Supplement: S1 File — Verb frequency list. (DOCX) [file pone.0322341.s001.docx]

**Supporting Information**

**S1 Appendix. Experimental Materials**

*Active/Passive sentences*

Two versions of each experimental sentence were created, corresponding to the experimental conditions outlined in example (1) below.

1a. Wanita itu menemui kemarin seorang pria di stasiun kereta.

1b. Wanita itu ditemui kemarin oleh pria di stasiun kereta.

(A woman meets/’is met’ yesterday (by) a man in the train station.)

| 2 | Polisi itu menembak langsung seorang perampok di malam hari |
| --- | --- |
| 3 | Tahanan itu meneror terus seorang penjahat di rutan |
| 4 | Balita itu menyiksa terus seekor kucing saat bermain |
| 5 | Pria itu membekuk langsung seorang pencuri di rumahnya |
| 6 | Penjahat itu menghajar terus seorang penculik dari rutan |
| 7 | Pria itu menangkap langsung seorang penjahat saat kejar-kejaran |
| 8 | Tentara itu mengamankan langsung seorang polisi saat berbuat onar |
| 9 | Pria itu menggemari sekali seorang wanita dari kantornya |
| 10 | Wanita itu menyela terus seorang pria saat pidato |
| 11 | Wanita itu menghubungi langsung seorang pria di siang hari |
| 12 | Wanita itu memarahi terus seorang pemuda saat merokok |
| 13 | Pemuda itu meminati sekali seorang wanita di kampusnya |
| 14 | Staf itu memberhentikan kemarin seorang pria karena perbuatannya |
| 15 | Staf itu mengirim kemarin seorang pegawai ke sebuah konferensi |
| 16 | Perawat itu menyuntik langsung seorang dokter di rumah sakit |
| 17 | Pegawai itu menguji terus seorang staf dari pagi hari |
| 18 | Pria itu memajang kemarin seorang pemuda yang mendapatkan penghargaan |
| 19 | Pemuda itu menugaskan kemarin seorang pegawai pada sore hari |
| 20 | Sekelompok petarung itu mengeroyok terus sekumpulan perusuh dari sekolah lain |
| 21 | Pria itu menculik kemarin seorang pemuda dari rumahnya |
| 22 | Wanita itu menemani kemarin seorang sahabat dari sekolahnya |
| 23 | Pria itu memulangkan kemarin seorang staf dari kantornya |
| 24 | Pemuda itu membacok kemarin seorang pria di jalanan |
| 25 | Pegawai itu menangani langsung seorang staf tentang pemberhentiannya |
| 26 | Polisi itu memeriksa kemarin seorang tentara di perbatasan |
| 27 | Polisi itu menahan langsung seorang tentara akibat tindak kriminal |
| 28 | Pria itu menyapa langsung seorang wanita di bandara |
| 29 | Wanita itu menjumpai kemarin seorang pria di kebun binatang |
| 30 | Wanita itu mewawancarai langsung seorang pria di kantor |
| 31 | Dokter itu merawat kemarin seorang perawat di klinik |
| 32 | Pemuda itu mengasuh terus seorang perempuan yang sudah lama dikenalnya |
| 33 | Polisi itu mewajibkan terus seorang tentara untuk membuat laporan |
| 34 | Pria itu memecat langsung seorang wanita karena tindakannya |
| 35 | Wanita itu meminjami langsung seorang pria uang untuk berobat |
| 36 | Wanita itu meneliti terus seorang pria mengenai kesehatannya |
| 37 | Dokter itu memvonis kemarin seorang hakim pada siang hari |
| 38 | Hakim itu menjerat langsung seorang pengacara karena kasus suap |
| 39 | Pria itu menyiagakan kemarin seorang pemuda untuk jaga malam |
| 40 | Wanita itu mengizinkan langsung seorang pria untuk mengambil cuti |
| 41 | Pria itu menamakan kemarin seorang wanita sebagai pahlawan negara |
| 42 | Wanita itu menyebut langsung seorang pria dalam pidatonya |
| 43 | Pria itu memfitnah terus seorang wanita di media sosial |
| 44 | Wanita itu membohongi terus seorang pria saat berdiskusi |
| 45 | Polisi itu menerjunkan langsung seorang tentara saat negosiasi dengan penculik |
| 46 | Pria itu mempermalukan terus seorang wanita di siaran televisi |
| 47 | Penjahat itu membantai kemarin seorang perampok dalam pertarungan |
| 48 | Wanita itu menuduh terus seorang pria sebagai pencuri |
| 49 | Pria itu menganiaya terus seorang wanita dalam rumah tangga |
| 50 | Wanita itu melarang terus seorang pria untuk keluar rumah |

Approximate translation of the materials – please note that the materials in Indonesian do not contain number information (i.e. noun plurality), gender (policeman/woman or he/she), definiteness (i.e. articles), tense, and aspect. So, a literal translation of (2) would be ‘police shoot immediately robber at night’.

| 2 | The police immediately shoots the robber at night |
| --- | --- |
| 3 | The prisoner continuously terrorizes the criminal in prison |
| 4 | The toddler continuously torments the cat while playing |
| 5 | The man immediately captures the person at home |
| 6 | The criminal continuously beats the kidnapper from the prison |
| 7 | The man immediately catches the person during a chase |
| 8 | The soldier immediately the police when he/she makes trouble |
| 9 | The man very much likes the woman from his office |
| 10 | The woman continuously interrupts the man during the speech |
| 11 | The woman immediately contacts the man during the day |
| 12 | The woman continuously scolds the young man while smoking |
| 13 | The young man is very much interested in the woman in his campus |
| 14 | The staff yesterday dismisses the man for his actions |
| 15 | The staff yesterday sends employees to a conference |
| 16 | The nurse immediately injects the doctor in the hospital |
| 17 | The worker continuously tests the staff from the morning |
| 18 | The man yesterday displays the young man who got the award. |
| 19 | The young man yesterday assigns employees in the afternoon |
| 20 | The group continuously gangs up on a mob from another school |
| 21 | The man yesterday kidnaps the youth from his house |
| 22 | The woman yesterday accompanies the friend from school |
| 23 | The man yesterday sends the staff home from his office |
| 24 | The young man yesterday slashes the man on the street |
| 25 | The worker immediately handled the staff regarding his/her dismissal |
| 26 | The police yesterday checks the soldier in the border |
| 27 | The police immediately arrests the soldier for his/her actions |
| 28 | The man immediately greets the woman at the airport |
| 29 | The woman yesterday meets the man in the zoo |
| 30 | The woman immediately interviews the man in the office |
| 31 | The doctor yesterday treats the nurse in the clinic |
| 32 | The young man continuously takes care of the woman he has known for a long time |
| 33 | The police continuously obliges the soldier to make a report |
| 34 | The man immediately fires the woman because of her actions |
| 35 | The woman immediately lends the man money for treatment |
| 36 | The woman continuously researches the man about his health |
| 37 | The doctor yesterday sentences (for a serious disease) the judge at noon |
| 38 | The judge immediately charges the lawyer for his/her involvement in the bribery case |
| 39 | The man yesterday alerts the young man to go on a night watch |
| 40 | The woman immediately allows the man to take time off |
| 41 | The man yesterday named the woman as a national hero |
| 42 | The woman immediately mentions the man in her speech |
| 43 | The man continuously slanders women on social media |
| 44 | The woman continuously lies to the man in the discussion |
| 45 | The police immediately fields the soldier during negotiations with the kidnappers |
| 46 | The man continuously humiliates the woman on live television |
| 47 | The villain yesterday slaughters the robber in a fight |
| 48 | The woman continuously accuses the man of being a thief |
| 49 | The man continuously molests the woman in their household |
| 50 | The woman continuously forbids the man to leave the house |

**S2 Appendix. Verb Frequency List**

S1 Table. Verb frequency list

|  | **Active** | **Passive** | **AFC** | **PFC** | **Afreq** | **Pfreq** | **English** |
| --- | --- | --- | --- | --- | --- | --- | --- |
| 1 | menembak | ditembak | 11 | 11 | 17922 | 13270 | shoot |
| 2 | meneror | diteror | 15 | 15 | 1455 | 1021 | terrorize |
| 3 | menyiksa | disiksa | 12 | 12 | 6648 | 6452 | torture |
| 4 | membekuk | dibekuk | 13 | 13 | 3146 | 5230 | capture/defeat |
| 5 | menghajar | dihajar | 13 | 13 | 4029 | 3795 | beat up |
| 6 | menangkap | ditangkap | 9 | 9 | 71298 | 77804 | capture |
| 7 | mengamankan | diamankan | 10 | 10 | 47758 | 25803 | secure |
| 8 | menggemari | digemari | 14 | 12 | 2748 | 10645 | be fond of |
| 9 | menyela | disela | 15 | 14 | 1104 | 2362 | interrupt |
| 10 | menemui | ditemui | 9 | 9 | 58956 | 82273 | meet |
| 11 | menghubungi | dihubungi | 9 | 9 | 91777 | 55632 | contact/call |
| 12 | memarahi | dimarahi | 13 | 13 | 3140 | 3042 | scold |
| 13 | meminati | diminati | 14 | 10 | 1542 | 29005 | be interested in |
| 14 | memberhentikan | diberhentikan | 13 | 12 | 5109 | 10026 | dismiss/lay off |
| 15 | mengirim | dikirim | 9 | 9 | 64365 | 69088 | send |
| 16 | menyuntik | disuntik | 15 | 14 | 1195 | 2429 | inject |
| 17 | menguji | diuji | 10 | 10 | 31037 | 25641 | test |
| 18 | memajang | dipajang | 13 | 12 | 4382 | 6775 | display |
| 19 | menugaskan | ditugaskan | 13 | 11 | 5300 | 14580 | assign |
| 20 | mengeroyok | dikeroyok | 15 | 14 | 1258 | 2830 | gang up on |
| 21 | menculik | diculik | 13 | 12 | 3186 | 6073 | kidnap |
| 22 | menemani | ditemani | 11 | 11 | 20606 | 14508 | accompany |
| 23 | memulangkan | dipulangkan | 13 | 12 | 4440 | 8380 | repatriate/return home |
| 24 | membacok | dibacok | 15 | 15 | 1218 | 1006 | slash |
| 25 | menangani | ditangani | 9 | 9 | 89803 | 51662 | handle |
| 26 | memeriksa | diperiksa | 9 | 9 | 61654 | 61640 | check |
| 27 | menahan | ditahan | 9 | 9 | 88180 | 50600 | detain |
| 28 | menyapa | disapa | 11 | 11 | 14141 | 22391 | greet |
| 29 | menjumpai | dijumpai | 12 | 10 | 8243 | 25800 | find |
| 30 | mewawancara | diwawancara | 16 | 14 | 425 | 2757 | interview |
| 31 | merawat | dirawat | 10 | 10 | 32224 | 39845 | treat/nurse |
| 32 | mengasuh | diasuh | 13 | 13 | 5889 | 4574 | take care of/nurture |
| 33 | mewajibkan | diwajibkan | 11 | 10 | 16803 | 28013 | oblige/necessitate |
| 34 | memecat | dipecat | 13 | 11 | 5898 | 14548 | fire (from a job) |
| 35 | meminjami | dipinjami | 17 | 16 | 301 | 411 | lend |
| 36 | meneliti | diteliti | 10 | 10 | 31213 | 25947 | research/investigate |
| 37 | memvonis | divonis | 13 | 11 | 4095 | 14157 | sentence/convict |
| 38 | menjerat | dijerat | 12 | 11 | 9830 | 12332 | ensnare (usually by law) |
| 39 | menyiagakan | disiagakan | 14 | 13 | 2475 | 3696 | alert |
| 40 | mengizinkan | diizinkan | 11 | 11 | 17842 | 15633 | allow/permit |
| 41 | menamakan | dinamakan | 13 | 10 | 5329 | 31583 | name |
| 42 | menyebut | disebut | 8 | 7 | 124627 | 342595 | mention |
| 43 | memfitnah | difitnah | 14 | 14 | 2638 | 1517 | slander/defame |
| 44 | membohongi | dibohongi | 14 | 14 | 2705 | 2287 | lie/deceive |
| 45 | menerjunkan | diterjunkan | 13 | 13 | 4465 | 5041 | mobilize/drop |
| 46 | mempermalukan | dipermalukan | 13 | 13 | 3475 | 3354 | humiliate/embarrass |
| 47 | membantai | dibantai | 13 | 13 | 3784 | 3246 | slaughter (usually in sports) |
| 48 | menuduh | dituduh | 11 | 11 | 22518 | 18108 | accuse |
| 49 | menganiaya | dianiaya | 13 | 13 | 5326 | 5450 | bully/mistreat |
| 50 | melarang | dilarang | 9 | 9 | 58153 | 55937 | prohibit |

AFC: active verb frequency class

PFC: passive verb frequency class

Afreq: active verb token frequency

Pfreq: passive verb token frequency

English: translation of the verbs
