# Supplementary material for: Registered Report: Neural correlates of thematic role assignment for passives in Standard Indonesian
Source: PLoS One. 2025 May 13;20(5):e0322341. doi: 10.1371/journal.pone.0322341 (PMC12074587; doi:10.1371/journal.pone.0322341)
Supplement: S2 File — S2 Fig. ERP (electrode P7) at the verb. S3 Fig. ERP (electrode P7) at NP2. S4 Fig. Topographic plot at the NP2 for the 500–700ms time window (passive-active). S5 Fig. Topographic plot at the Verb for the whole epoch (passive-active). S6 Fig. Topographic plot at the NP2 for the whole epoch (passive-active). S7 Fig. ERP plots at the verb, part 1 (passive-active). S8 Fig. ERP plots at the verb, part 2 (passive-active). S9 Fig. ERP plots at the verb, part 3 (passive-active). S10 Fig. ERP plots at the verb, part 4 (passive-active). S11 Fig. ERP plots at the NP2, part 1 (passive-active). S12 Fig. ERP plots at the NP2, part 2 (passive-active). S13 Fig. ERP plots at the NP2, part 3 (passive-active). S14 Fig. ERP plots at the NP2, part 4 (passive-active). (DOCX) [file pone.0322341.s002.docx]

**Supporting Information**

**S3 Appendix. Supplementary analysis with stricter exclusion criteria**

Our originally pre-registered criteria for participant inclusion turned out to be very strict, so in the end we had to relax the inclusion criteria (deviating from the original registered report protocol) in order to collect 50 participants’ worth of usable data. Here, we report an analysis from the 31 participants who met the original, strict inclusion criteria from a total of 60 participants tested.

We utilized cluster-based permutation of the whole epoch. The difference between active and passive sentences on the verb was not significant (*p*=.195). However, we observed an ERP on the NP2 (*p*=.013) in the form of a broadly distributed and sustained positive shift. The clusters started early at about 50ms and ended at around 700ms (Figure S1).


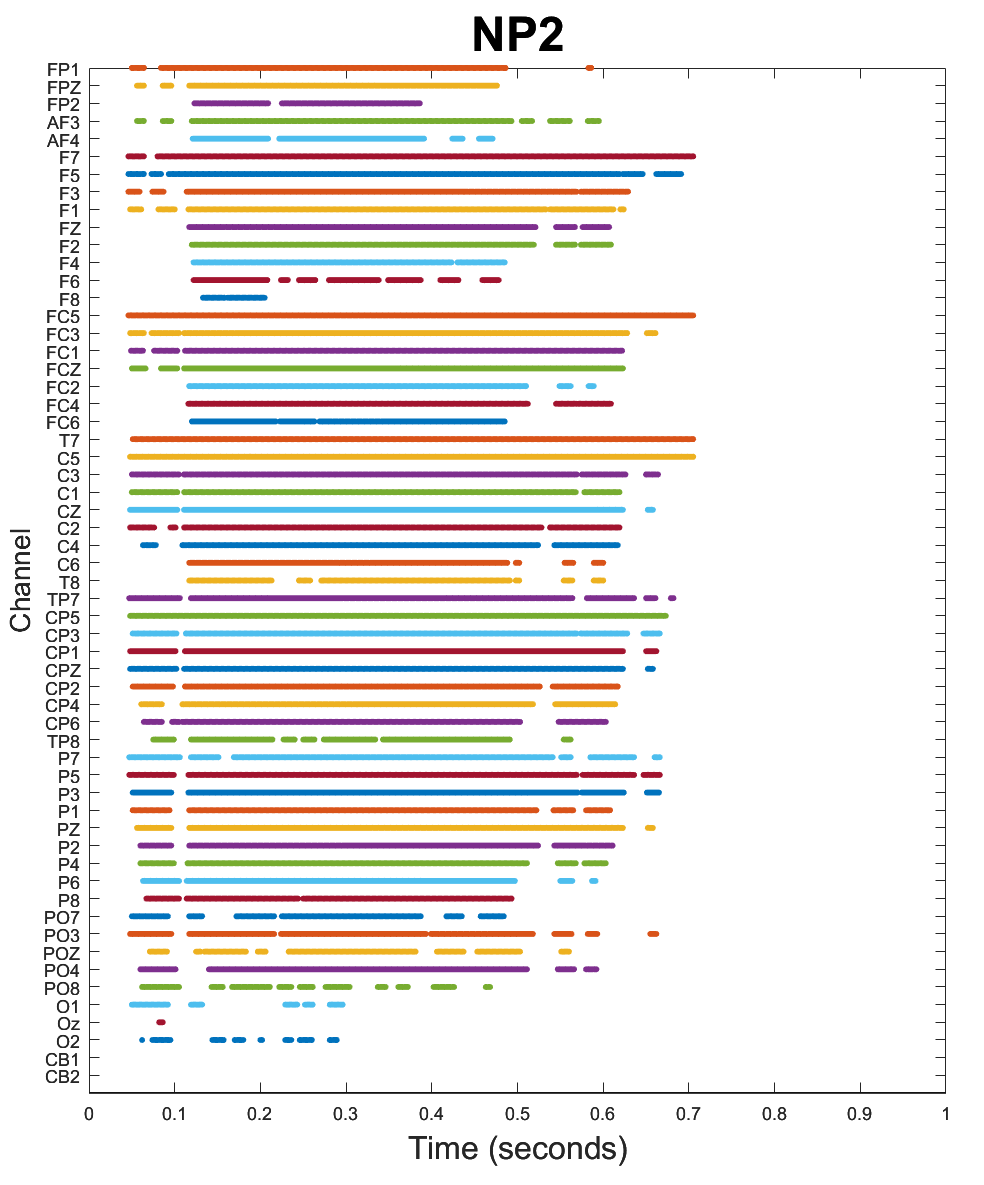


S1 Fig. Raster plot showing which data points were included in the permutation test for the whole epoch at NP2.

ERP plots at the verb and NP2 (Figure S2 and Figure S3) can be found below. The channel displayed for Figure S3 was one of the channels where the permutation test found significant clusters (P7). While the analysis showed a sustained effect at NP2, the time window of interest for a positivity was the P600 time window, and as such Figure S4 provides topographic plots of the difference between passive and active conditions at NP2 at the 500 to 700ms time window.


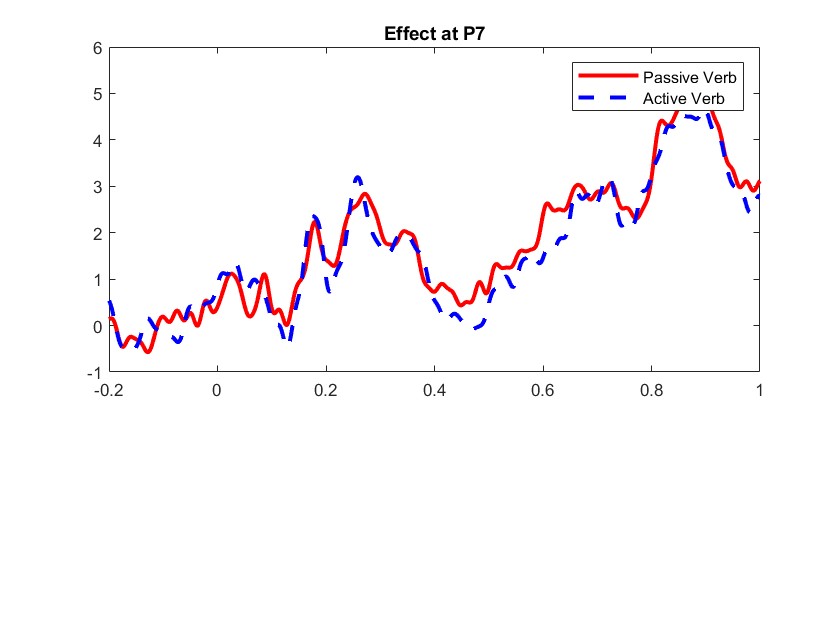


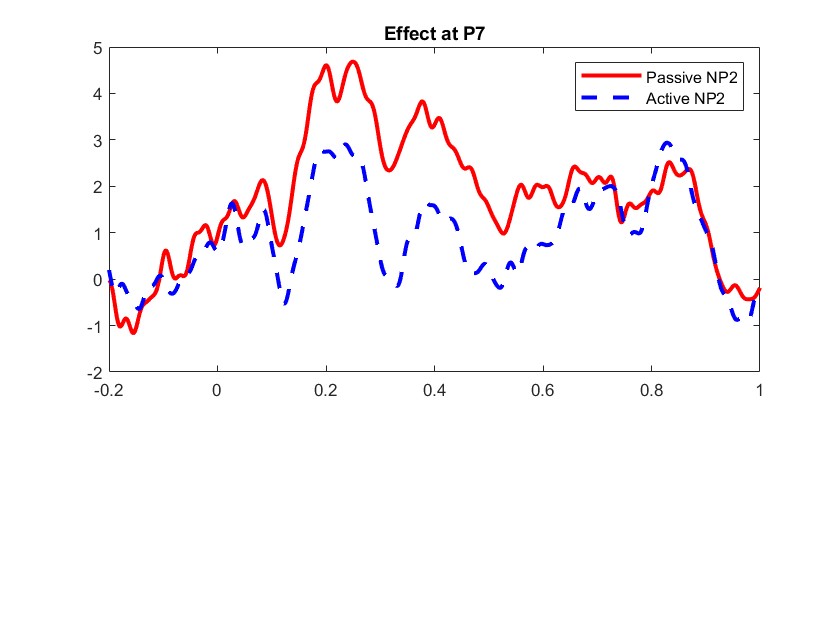
S2 Fig. ERP (electrode P7) at the verb

S3 Fig. ERP (electrode P7) at NP2

There seems to be a sustained, widespread positivity at the NP2 region, which mirrors our main analysis and can be observed at both the topographic maps (Figure S4) and the ERP plot (Figure S3). From visual observation, the ERP plot of the verb (Figure S2) seems to show a small positive wave peaking just after 600ms. However, this visual observation is not confirmed by our whole-epoch analysis.


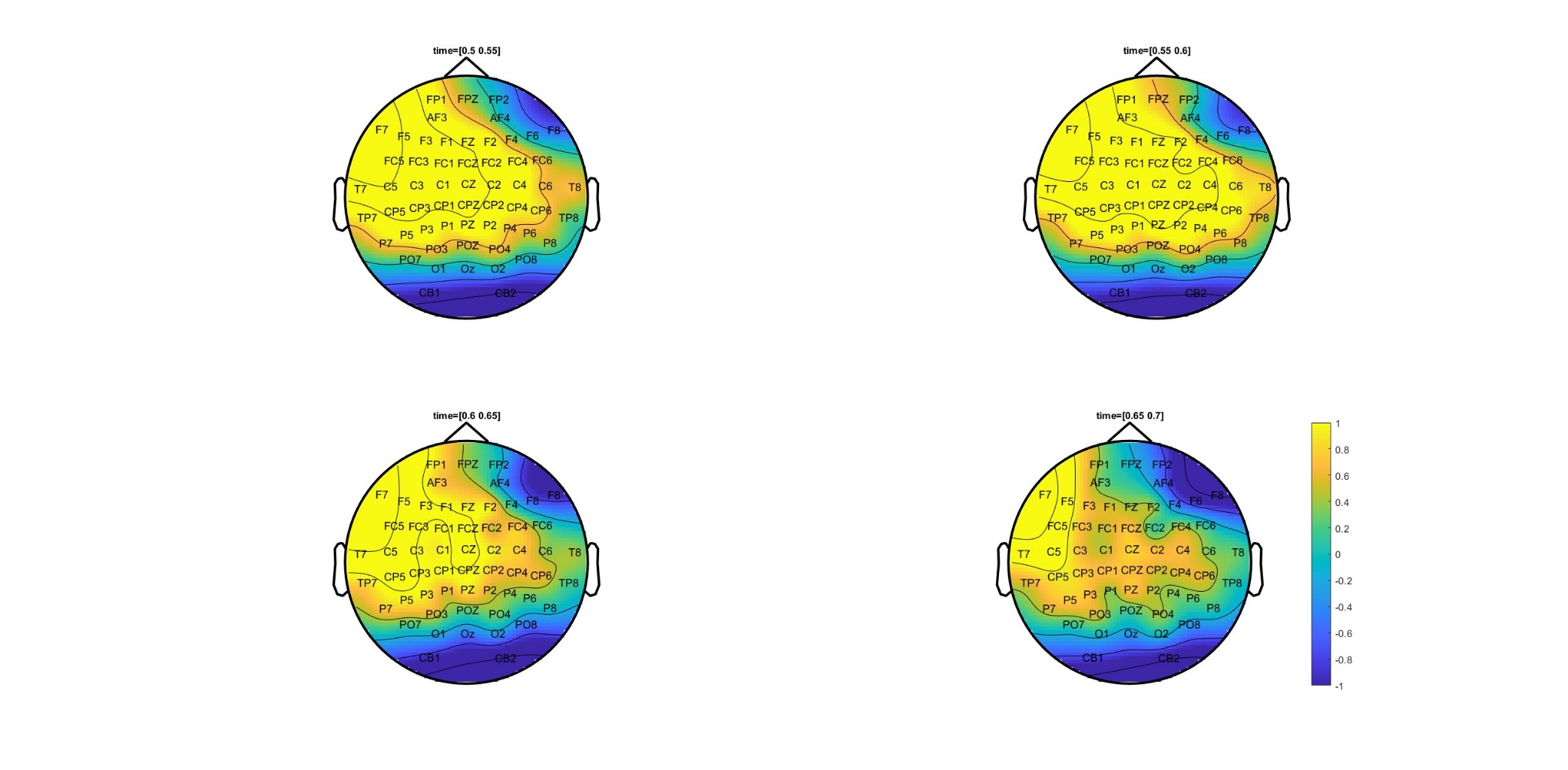


S4 Fig. Topographic plot at the NP2 for the 500 to 700ms time window (passive-active)

**S4 Appendix. ERP plots for all channels and topographic plots for the full epoch in all conditions**

**
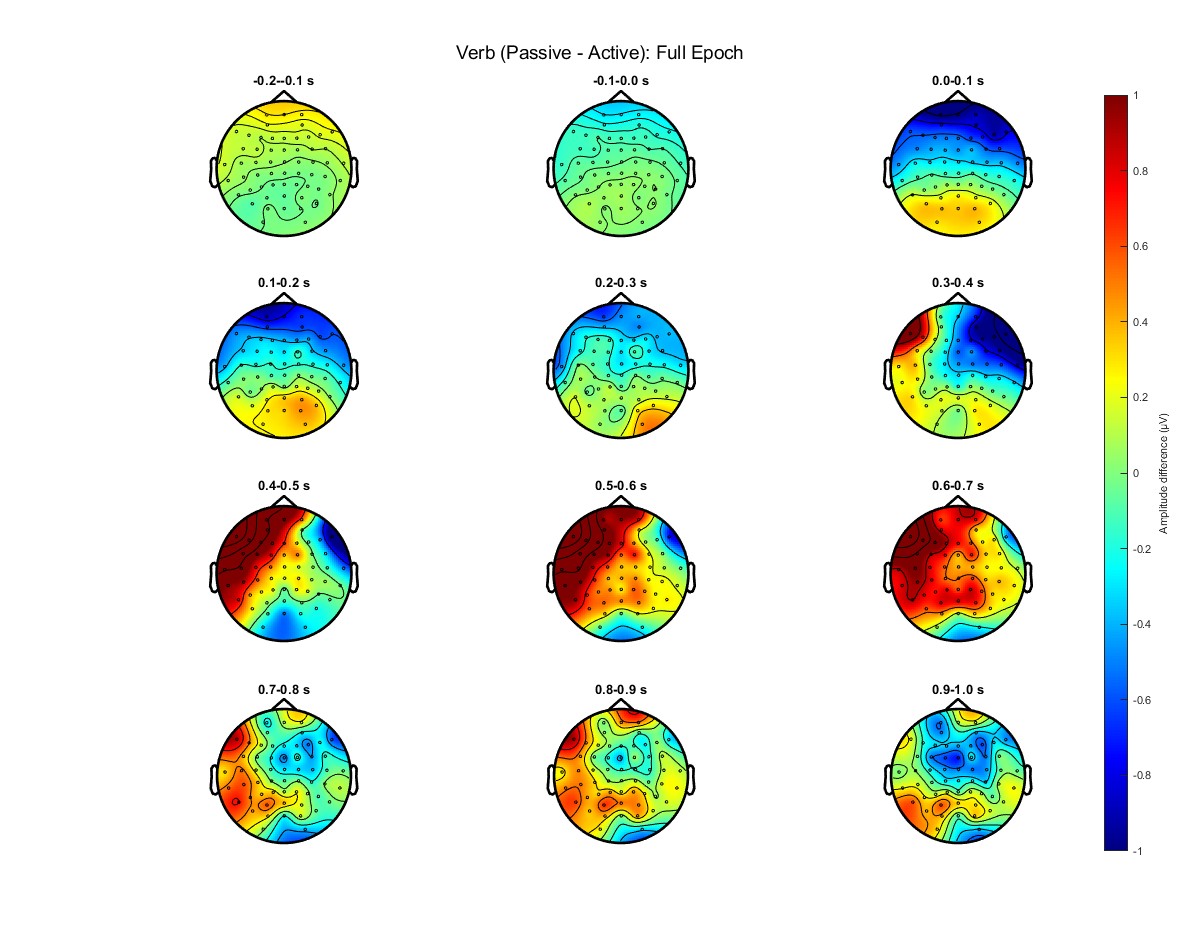
**

S5 Fig. Topographic plot at the Verb for the whole epoch (passive-active)


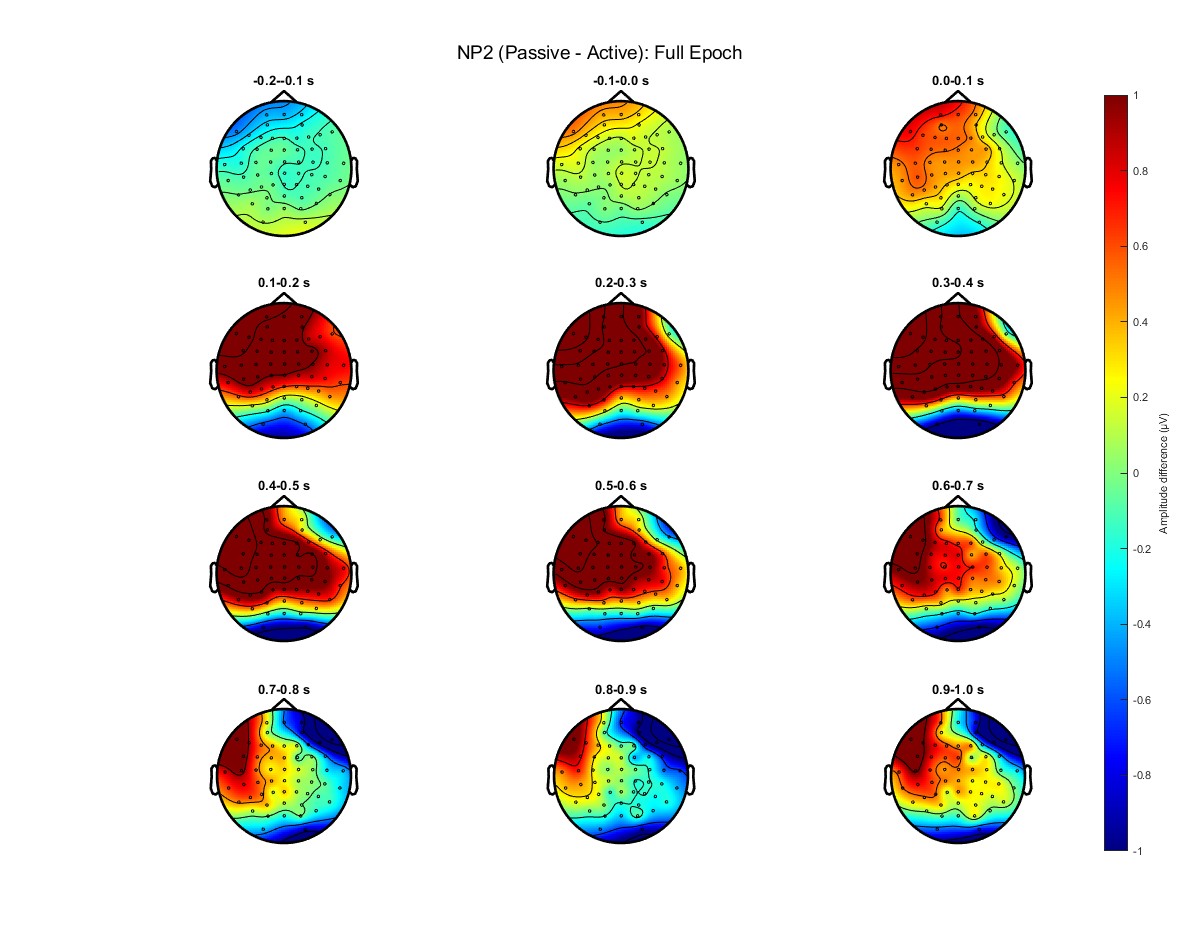


S6 Fig. Topographic plot at the NP2 for the whole epoch (passive-active)


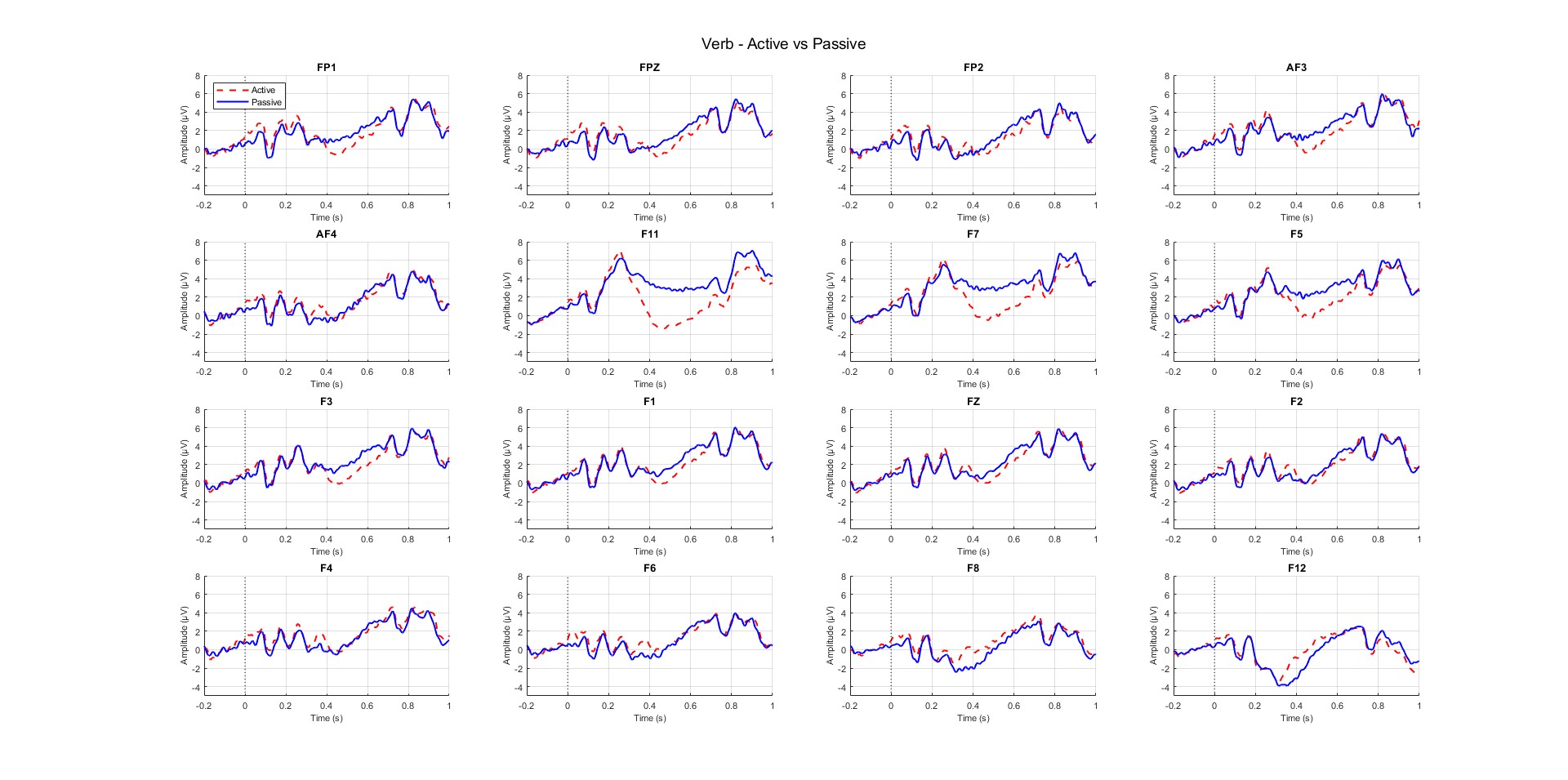


S7 Fig. ERP plots at the verb, part 1 (passive-active)


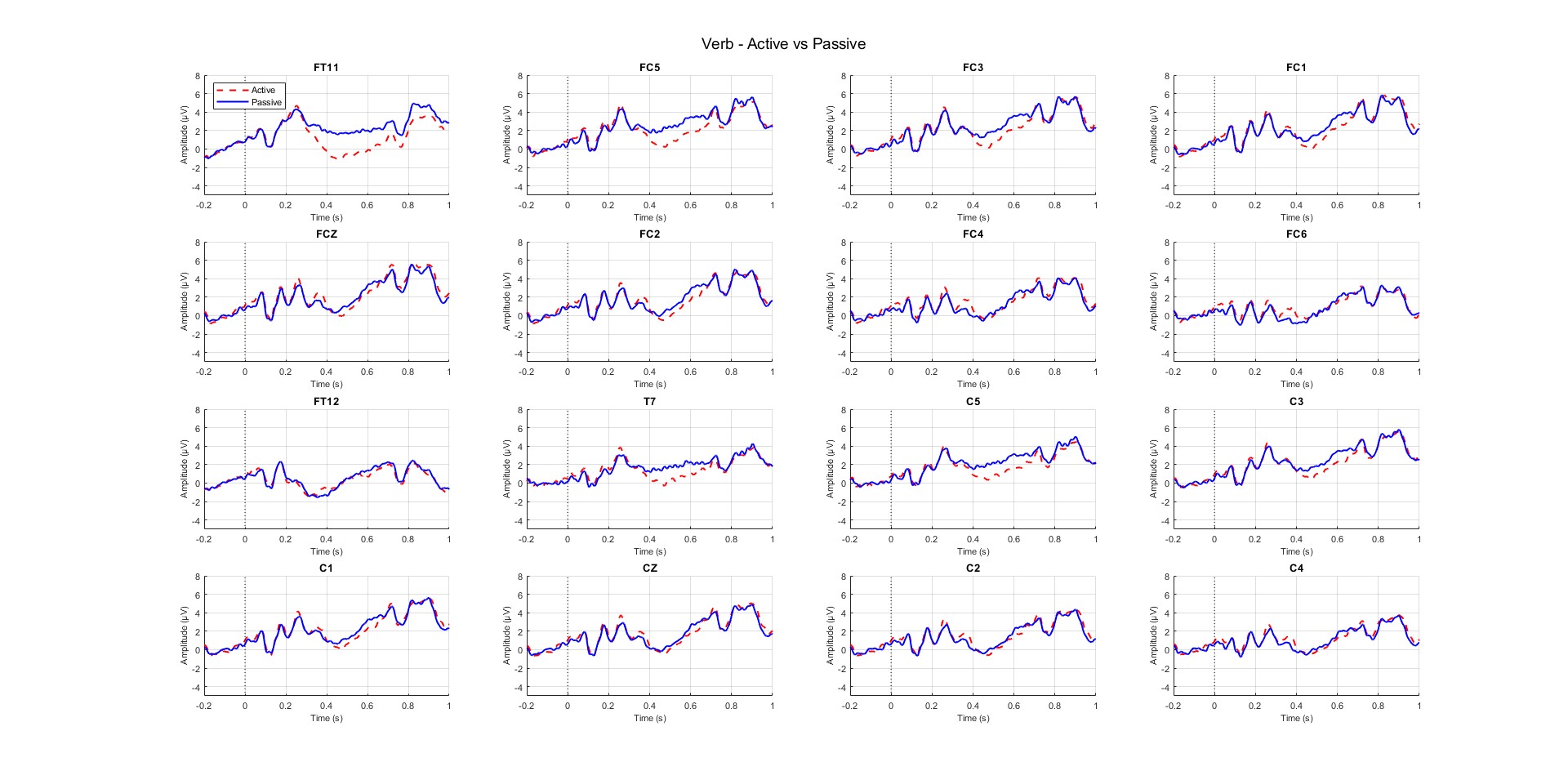


S8 Fig. ERP plots at the verb, part 2 (passive-active)


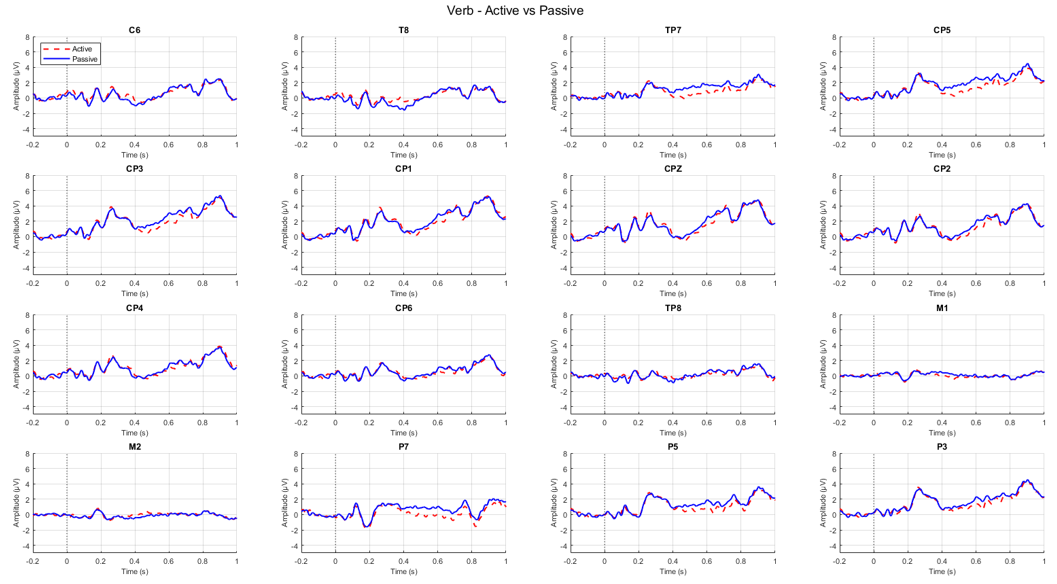


S9 Fig. ERP plots at the verb, part 3 (passive-active)


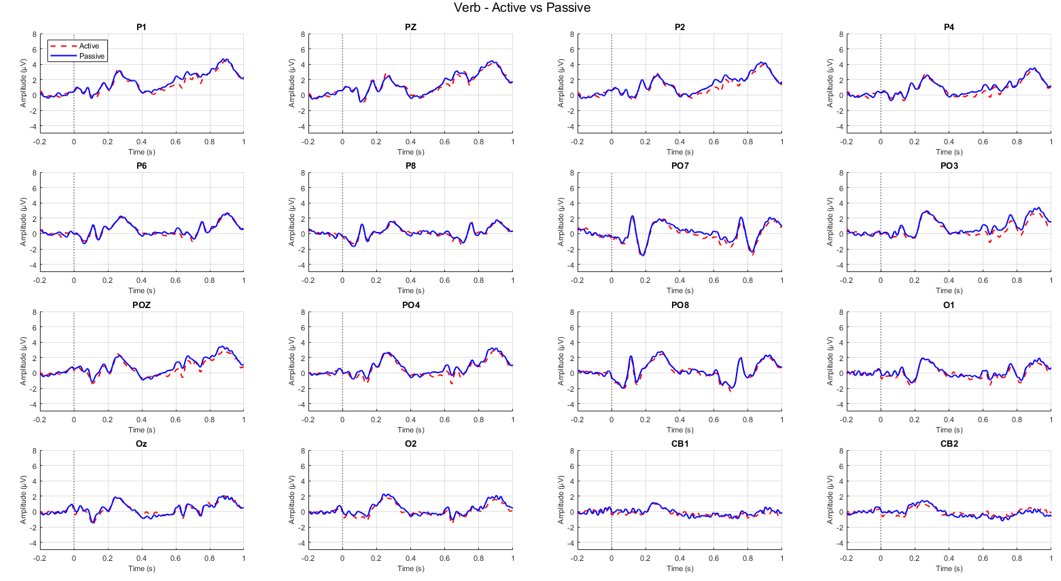


S10 Fig. ERP plots at the verb, part 4 (passive-active)


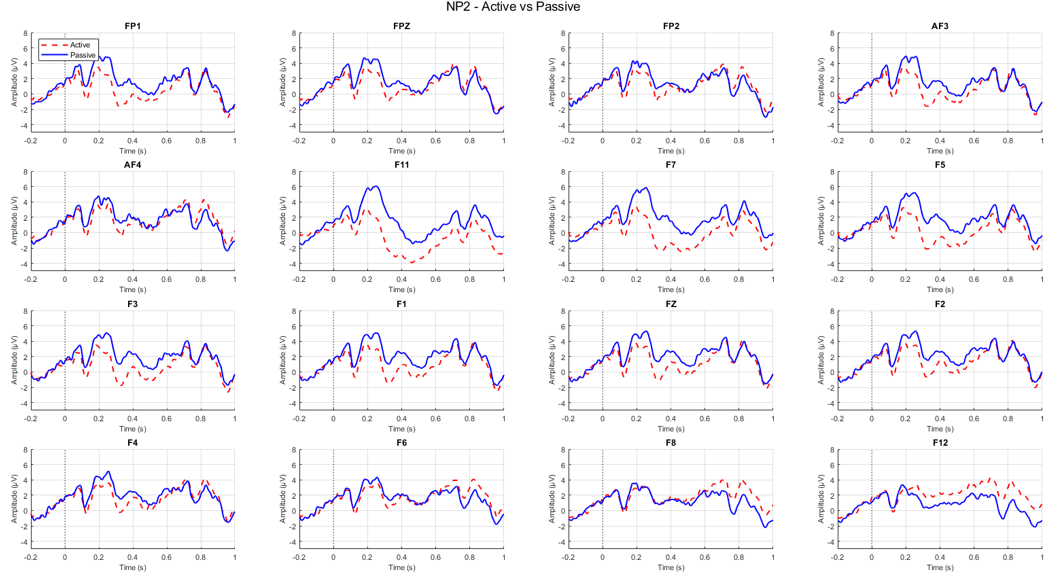


S11 Fig. ERP plots at the NP2, part 1 (passive-active)


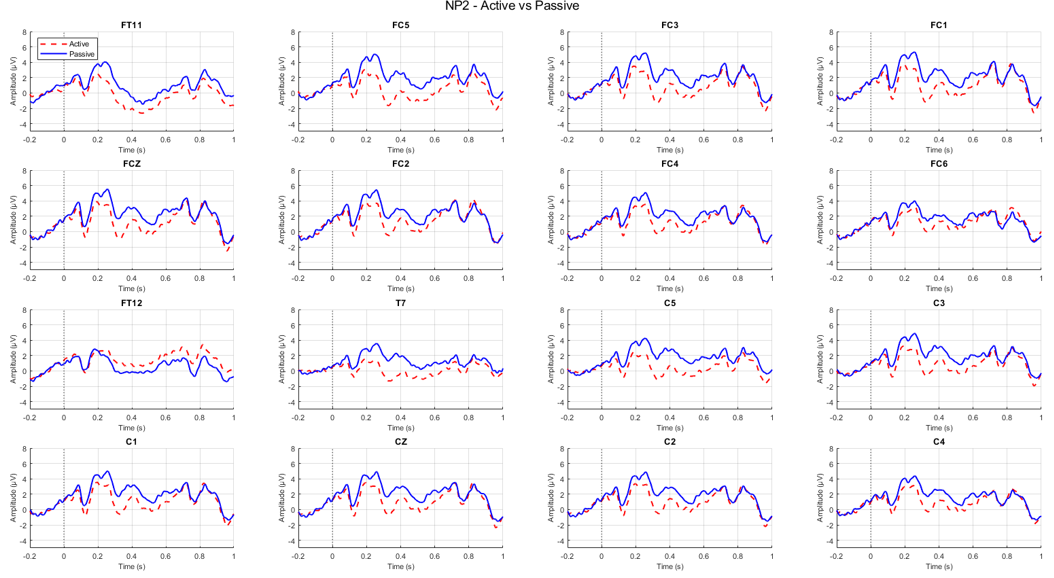


S12 Fig. ERP plots at the NP2, part 2 (passive-active)


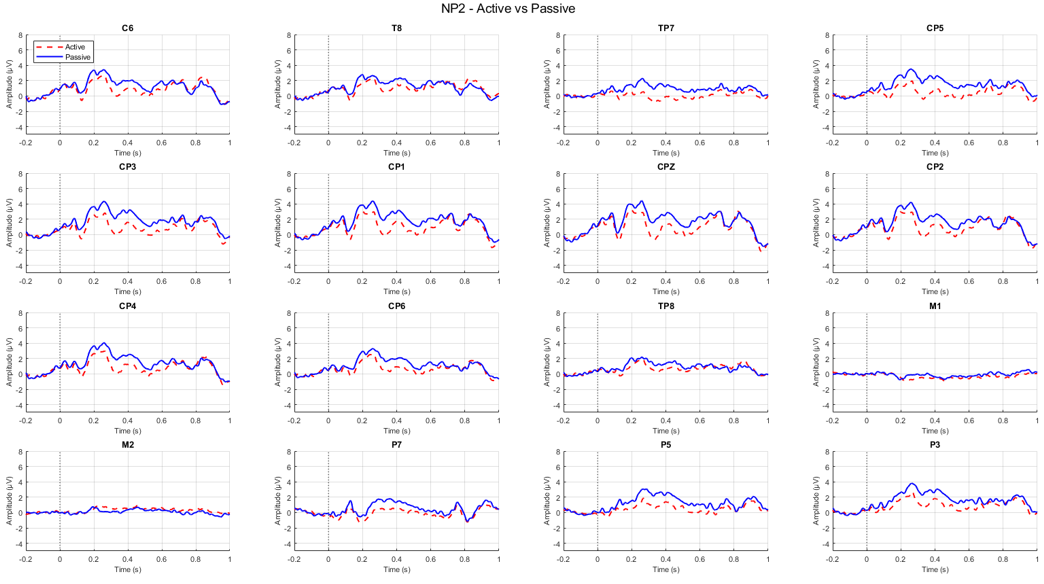


S13 Fig. ERP plots at the NP2, part 3 (passive-active)


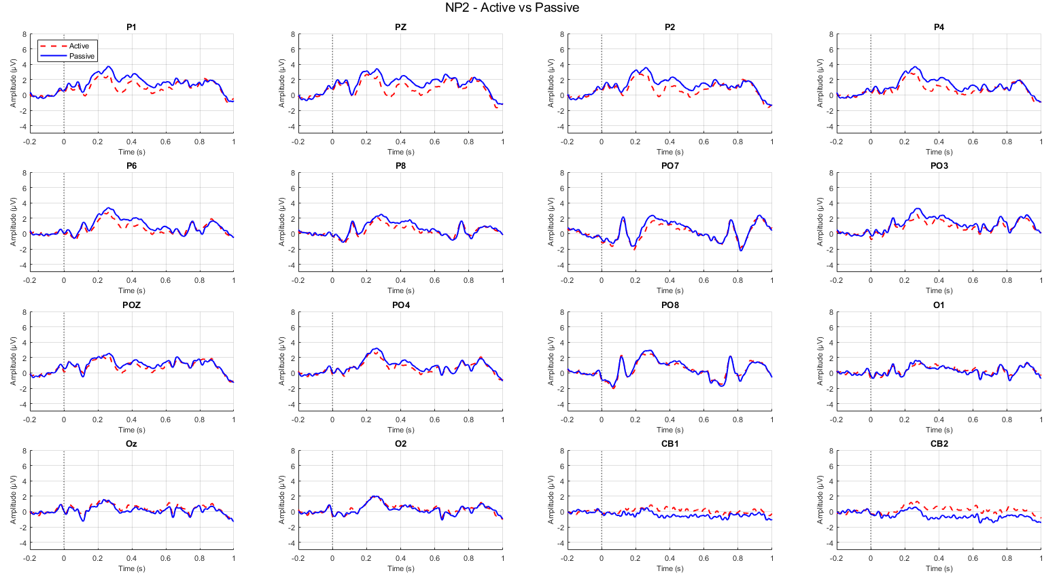


S14 Fig. ERP plots at the NP2, part 4 (passive-active)
